# Hospital-at-Home for South Asian Communities in British Columbia, Canada: Qualitative Interview Study

**DOI:** 10.2196/79675

**Published:** 2026-01-05

**Authors:** Emma Wong, Mahabhir Kandola, Kamal Arora, Harroop Sharda, Roman Deol, Mary Jung, Robert Paquin, Maria Montenegro, Megan MacPherson

**Affiliations:** 1 Department of Virtual Health, Fraser Health, 13450 102 Ave, Surrey, BC, V3T0H1, Canada, 1 6045616605; 2 British Columbia Institute of Technology, Burnaby, BC, Canada; 3 School of Health and Exercise Sciences, University of British Columbia, Kelowna, BC, Canada; 4 King's College London, London, United Kingdom; 5 Department of Occupational Science and Occupational Therapy, Faculty of Medicine, University of British Columbia, Vancouver, BC, Canada

**Keywords:** telemedicine, South Asian people, qualitative research, health equity, Hospital-at-Home

## Abstract

**Background:**

South Asian communities in Canada face significant disparities in access to health care and experience higher rates of chronic conditions such as cardiovascular disease, diabetes, and hypertension. Hospital-at-Home services have the potential to improve access and outcomes, yet little is known about how these services are perceived and experienced by South Asian patients and caregivers. Understanding both barriers and facilitators is critical for culturally responsive implementation.

**Objective:**

This study aimed to explore the experiences of South Asian community members with in-person hospital care and their perceptions, attitudes, and expectations regarding virtual Hospital-at-Home services, with the goal of identifying culturally tailored strategies to improve access, quality, and satisfaction.

**Methods:**

A qualitative study using semistructured interviews was conducted with 20 South Asian community members in the Fraser Health region in British Columbia, Canada. Interviews explored experiences with in-person hospital care, perceptions of a virtual hospital service (also known as Hospital-at-Home), and recommendations for enhancing awareness and accessibility. Interviews were audio-recorded, transcribed, and analyzed thematically to identify key patterns in perceptions, experiences, and needs.

**Results:**

Participants described multiple systemic barriers to in-person hospital care, including long wait times, overcrowding, transportation challenges, and difficulty navigating the health system. Cultural and religious needs, such as gender-concordant care and culturally appropriate food, were frequently unmet, while language-concordant care and family involvement were critical to positive experiences. Discrimination and assumptions based on ethnicity or age further shaped perceptions of care. Virtual hospital services were valued for convenience, comfort, reduced exposure to hospital-acquired infections, and support for family involvement. However, participants raised concerns about clinical quality, the absence of physical examinations, digital literacy, privacy, and home-based responsibilities. Acceptance varied by age, immigration status, and familiarity with technology. Participants emphasized the importance of culturally tailored outreach, leveraging community leaders, ethnic media, and peer testimonials to increase awareness and trust.

**Conclusions:**

South Asian patients and caregivers recognize both challenges in traditional hospital care and potential benefits of Hospital-at-Home services. Implementation strategies that address systemic barriers, integrate cultural and linguistic considerations, and engage trusted community networks are essential to improving equity, access, and satisfaction. Findings highlight the need for culturally responsive, patient-centered approaches in the design and delivery of virtual health services for racialized populations.

## Introduction

### Background

The South Asian population, which includes individuals with ethnic roots originating from Bangladesh, Bhutan, India, Maldives, Nepal, Pakistan, and Sri Lanka, constitutes the largest visible minority or racialized group in Canada [1]. South Asians in Canada face a higher prevalence of cardiovascular disease, diabetes, and hypertension compared to other racialized groups [2,3]. While research suggests that genetic predisposition is a partial contributor [4], the observed health disparities likely also result from social and environmental determinants of health, such as access to higher education, employment opportunities, food security, experiences of discrimination, and access to culturally appropriate health care services [5-7]. It is important to recognize that South Asian populations in Canada are highly diverse, encompassing a range of languages, religions, socioeconomic backgrounds, and migration histories, all of which influence health experiences and outcomes. Previous research has shown that South Asians encounter significant barriers in accessing health care resources for chronic disease prevention and management [8]. Given the inequities and disparities faced by South Asian community members within Canada, innovative care strategies are needed to enhance access to culturally relevant care and improve health outcomes.

### Virtual Health Care

Virtual health care, which uses remote technologies to deliver health services, has been identified as a key mechanism for improving access to care [9]. Specifically, virtual hospitals-at-home (commonly referred to as simply Hospital-at-Home) provide an alternative to traditional “brick and mortar” hospital admissions, allowing patients to receive the care they need from the comfort of their own home [10]. This care model has been increasingly used worldwide, accelerated by hospital capacity strains during the COVID-19 pandemic [11,12]. While admitted to a Hospital-at-Home, patients are monitored remotely using digital devices that transmit data to their multidisciplinary care team [10] and have daily virtual consultations via telephone or video, supplemented with home visits when necessary [10].

Research has shown that Hospital-at-Home models are both feasible and effective across various conditions [13]. Studies report that patients opt into virtual hospitals at high rates, and that this approach delivers high-quality care with fewer complications (eg, shorter hospital stays, lower incidence of delirium, and reduced rate of falls [14]). Patients and caregivers frequently express higher satisfaction with home-based care, citing increased comfort, privacy, and family engagement as key benefits [13].

Beyond individual benefits, Hospital-at-Home can alleviate system-wide pressures by reducing wait times, freeing up hospital bed capacity, and expanding access to care [15]. Despite these advantages, challenges remain. Some studies have noted higher readmission risk for patients with complex medical conditions, as well as increased caregiver burden in home settings [13]. Additionally, concerns about communication barriers and the need for structured care coordination highlight areas where further improvements are needed to ensure equitable and effective implementation of Hospital-at-Home [13].

Given the potential of virtual hospitals to enhance access to care, particularly for underserved populations, it is critical to understand and address the barriers to their adoption and use. This is especially important for racialized communities, such as South Asians in Canada, who already experience health disparities and face unique challenges in accessing virtual health services [16].

### Virtual Health Use in South Asian Communities

While virtual care has the potential to alleviate barriers experienced by South Asian communities [17] in Canada, and research suggests positive attitudes toward integrating virtual services [16], significant barriers still exist for accessing these services. Studies have shown that age, gender, income, education, and language are associated with the ability to use health-related technologies among Panjabi-speaking (also known as “Punjabi”) South Asian individuals in Canada [18] and that language preferences, education, age, and sex predict the use of virtual care among South Asians in Alberta [19]. In both studies, older adults, women, those with a preference for non-English communication, and those with lower educational levels were less likely to engage with virtual care [18,19]. A recent community-based participatory action research project in Surrey, British Columbia, Canada further supported these findings [16]. Through focus groups and photo voice activities, participants identified concerns about their ability to navigate new technologies as they age, gendered responsibilities limiting women’s access to technology, and communication difficulties due to language barriers, literacy levels, and digital literacy [16].

Although virtual care is designed to support disease prevention and self-management, these services are often developed without equity in mind, are not culturally responsive, and are generally underused by South Asian populations in Canada [16]. The divide in access and engagement with virtual care services could further exacerbate health disparities faced by South Asian communities [8]. Given the relative novelty of virtual hospitals, little is known about their acceptance among South Asian communities. Future virtual care models must address known barriers to ensure equitable access.

### Setting

Health care in British Columbia, Canada, is provided by a provincial health authority, 5 regional health authorities, and a First Nations health authority [20]. The Fraser Health Authority is the largest regional health authority, serving over 2 million people across 20 diverse and rapidly growing communities [21]. According to the 2021 Census, nearly 1 in 5 Fraser Health residents identifies as South Asian [22]. Fraser Health emphasizes understanding how religion, language, and cultural practices influence health to develop customized programs and services.

To better support South Asian communities, Fraser Health established the South Asian Health Institute (SAHI) in 2013, focusing on research, innovation, and evidence-based care [5]. Fraser Health’s Virtual Health team supports the development and implementation of new virtual care pathways across the region. One such pathway is the virtual hospital service. As Fraser Health serves 75% of South Asian residents in the province, it is essential to conduct patient-oriented research, or research done in direct partnership with patients, to understand what matters to South Asian communities when developing the virtual hospital [5].

### Research Objectives

This patient-oriented research study aims to engage South Asian community members through 3 primary objectives:

1. Capture and understand the emotional experiences, as well as the perceived barriers and facilitators, of members within South Asian communities in the context of both in-person and Hospital-at-Home systems.
2. Understand the lived experiences of South Asian community members in navigating the health care system and identify gaps in accessing care.
3. Identify opportunities for how Hospital-at-Home services can be implemented to improve the access and flow of care among South Asian communities.

## Methods

### Overview

This qualitative study used semistructured interviews to explore the experiences, perceptions, and attitudes of South Asian community members in British Columbia, Canada, toward in-person hospital care and the emerging Virtual Hospital-at-Home model. The study was guided by a patient-oriented research approach, prioritizing the perspectives of patients and caregivers to inform culturally relevant virtual care strategies.

This study was guided by a steering group including members from Progressive Intercultural Community Services (PICS) Society [23], Fraser Health’s Virtual Health department [24], Fraser Health’s SAHI [25], and Fraser Health’s Equity, Diversity, and Inclusion team [26], and academic partners from the University of British Columbia and King’s College London.

Interviews were guided by a pragmatic epistemology, which seeks to generate practical findings that can be readily applied in real-world settings while also tolerating multiple truths [27]. Although all South Asian community members may be exposed to the same care environments, everyone has unique experiences.

### Recruitment

Participants were recruited through convenience sampling using outreach by the PICS Society and the SAHI, and supplemented with community outreach efforts including social media, places of worship, and public libraries. The inclusion criteria were that participants must (1) identify as South Asian, (2) reside within the Fraser Health region, and (3) have been a patient in the hospital in the past year or have been a caregiver to someone in the hospital in the past year. Recruitment continued until thematic saturation was reached, when no new concepts emerged from subsequent interviews.

### Data Collection

South Asian community members were interviewed to understand their lived experiences navigating the virtual health care system. A topic guide, adapted from previous Virtual Health patient partner workshops, was revised with feedback from community members and edited by Fraser Health’s plain language team for clarity. The guide covered 2 primary domains: (1) experiences with in-person hospital care, including barriers, facilitators, and family involvement; and (2) perceptions of, and attitudes toward, the Virtual Hospital-at-Home model, including benefits, concerns, and strategies for community engagement. Interviews began with a screening question to classify participants as patients, caregivers, or both. A full interview topic guide is provided in Multimedia Appendix 1.

Thirty- to 60-minute, one-on-one interviews were conducted via Microsoft Teams, with the PICS Society providing technical support when needed to ensure participant access to the platform. To ensure accessibility, interpretation services were available in Panjabi, Hindi, Urdu, and Pashto. All interviews were audio-recorded with participant consent, transcribed verbatim, and translated where required by members of the research team. Interviews were conducted by EW and MK, who both had master’s degrees and worked as a “Research Assistant” and the “Research and Knowledge Translation Lead” for the Virtual Health department at the time of interviews. All interviewees had no prior relationships with those conducting the interviews.

### Data Analysis

Thematic analysis guided by the approach of Braun and Clarke [28] was conducted using NVivo (QSR International). First, transcripts were read repeatedly for familiarization. An initial set of open codes was inductively generated to capture salient features of the data. Codes were iteratively refined and grouped into higher-order categories. Themes were then developed through constant comparison across transcripts, paying attention to both convergent and divergent perspectives. Theme prevalence was assessed approximately by noting how many participants raised each theme, though exact tallies were not feasible given the conversational variability of interviews. Findings were also interpreted with attention to cultural safety and equity considerations relevant to South Asian communities in the Fraser Health region.

Coding was conducted by MK and cross-checked by MM; discrepancies were resolved by consensus. For multilingual interviews, translations were reviewed by bilingual team members to ensure conceptual equivalence. Final themes were reviewed and refined in consultation with the steering committee to enhance cultural validity. The steering committee did not influence the coding of raw data. Thematic saturation was achieved by the 17th interview; 3 additional interviews confirmed that no new themes emerged.

### Ethical Considerations

This study received approval from the Fraser Health Research Ethics Board (H23-03144). Interested participants were sent a consent form, the topic guide, and a video explaining the Hospital-at-Home to review beforehand. Informed consent for participation and interview recording was obtained verbally prior to the interview. To protect privacy and confidentiality, all transcripts were anonymized, and identifiers were removed prior to analysis. Data were stored securely on password-protected institutional servers. Participants were compensated with a US $14.86 gift card at the conclusion of their interview.

## Results

### Participant Characteristics

Twenty participants were interviewed (n=9 male, n=11 female). Participants were aged 19‐79 years, most commonly between 60‐69 years (6/20, 30%). Younger adults aged 19‐29 (n=5), 30‐39 (n=4), and 40‐49 (n=4) years were also represented, along with 1 participant in their 70s. Interviews were conducted in English (n=15), Panjabi (n=3), Pashto (n=1), and Hindi (n=1).

Most participants were originally from India (14/20, 70%), with others from Afghanistan (3/20, 15%), and smaller representations from Iraq (n=1), Pakistan (n=1), and India and Fiji (n=1). Half had lived in the community for more than 10 years (n=10); the remainder were more recent arrivals (n=4 lived in Canada <1 year, n=5 lived here 1‐3 years). Regarding immigration status, 9 were Canadian citizens and 11 were permanent residents.

Self-rated health was generally positive—6 participants (30%) rated their health as “very good,” 6 (30%) as “good,” and 8 (40%) as “average.” Six participants (30%) reported having a chronic condition. Participants included caregivers only (7/20, 35%), patients only (6/20, 30%), and both caregivers and patients (5/20, 25%). Two participants also contributed professional perspectives, working in newcomer services and health care.

All participants had access to a smartphone (20/20, 100%), while 11 (55%) had access to a tablet and 11 (55%) had access to a laptop. Ten participants (50%) had all 3 devices, while 8 (40%) had only a smartphone. Device use was frequent—14 (70%) used devices daily or multiple times per day, 4 (20%) used devices regularly but not daily, and 2 (10%) did not regularly use devices.

### Experiences of In-Person Hospital Care

#### Overview

Participants described a mix of challenges and positive experiences with in-person hospital care. Six overarching themes emerged: systemic barriers to accessing care, communication and language barriers, cultural and religious needs not met, family and caregiver roles, discrimination and bias, and positive experiences and gratitude. Experiences varied by age, gender, immigration status, and length of time in Canada. Older and more recently arrived participants reported more barriers than younger or Canadian-born participants.

#### Systemic Barriers to Accessing Care

Participants frequently reported challenges accessing timely and appropriate hospital services, including long wait times, overcrowding, transportation difficulties, and navigation challenges. These barriers contributed to frustration, fatigue, and inequitable access.

##### Long Wait Times

Wait times were among the most common challenges mentioned. Many participants described hours-long waits in emergency departments, sometimes extending overnight or across multiple days. As 1 participant shared, “we have waited with a broken wrist 28 hours” (P10, female, aged 60‐69 years, >10 years in community). Another recalled, “you have to wait maybe sometimes 10 hours or more than that and you have a critical illness” (P20, female, aged 60‐69 years, >10 years in community). Such prolonged waits were perceived as not only inconvenient but also unsafe when health concerns were urgent.

##### Overcrowding and Understaffing

Participants described hospitals as overcrowded and short-staffed, with patients left in hallways and nurses visibly stretched thin. One participant reported, “patients are all outside in the hallways. They don’t even have proper rooms” (P13, female, aged 60‐69 years, <1 year in community). Another observed, “sometimes you can see like the nurses are really tired, and then you see them overwhelmed with the amount of work” (P11, female, aged 19‐29 years, >10 years in community). These accounts reflect how structural resource constraints were felt by both patients and providers.

##### Transportation Difficulties

Transportation presented another layer of inequity, particularly for patients without private vehicles. Some relied on long bus rides with multiple transfers, which became especially challenging for older adults and those with mobility issues. One participant explained,

The nearest hospital is like a 45 minute transit ride, but the bus doesn’t go directly there. So I have to walk. For me as an able-bodied male, mid-20s, it’s fine. But...for my grandma who doesn’t drive and can’t speak much English, it’s very hard.

[P1, male, aged 20‐29 years, >10 years in community]

Another recalled, “I rely on HandyDART, and if that’s not available then my son would have to take time off work to drive me” (P17, female, aged 30‐39 years, >10 years in community). These logistical challenges shaped how and when participants sought hospital care.

##### Navigation Challenges for Newcomers

For newcomers to Canada, navigating the health system added further barriers. Several participants spoke about confusion around referrals, insurance, and entitlements. One reflected,

I took around a year’s time to understand what was MSP and how to access it. What is covered under it and what do I need other insurance for?

[P8, female, aged 30‐39 years, 1‐3 years in community]

Another highlighted misunderstanding about referral pathways:

You have to first go to a family doctor, and then if the problem is serious then you go to hospital. Many of the people don’t have that understanding.

[P6, male, aged 40‐49 years, >10 years in community]

Such experiences suggest that lack of system literacy can delay or complicate access to hospital care.

### Language and Communication

Communication challenges were central to participants’ accounts, particularly for older adults and newcomers with limited English proficiency, with many describing difficulties conveying symptoms, understanding instructions, or navigating interactions without the help of family. While some praised interpreters or language-concordant providers, gaps in communication were widely felt and often shaped the overall hospital experience.

#### Limited English Proficiency

Participants often highlighted the difficulty of explaining their health concerns in English. For example, one explained,

If I talk about my grandfather, they don’t know how to speak in English...if they are taking them alone into the rooms, they can’t express how they’re feeling or they can’t tell about their problems.

[P5, male, aged 19‐29 years, 1‐3 years in community]

Another noted, “when the other person is not from my community, the only hurdle I feel is the language” (P9, female, aged 30‐39 years, 4‐6 years in community). These challenges not only caused stress but also risked miscommunication in clinical encounters and increased reliance on family members acting as interpreters. One participant described, “she had a family member of hers that spoke on her behalf and communicated with the doctors” (P4, female, aged 60‐69 years, >10 years in community). Similarly, another shared, “mom didn’t speak English, so until I got there and started helping her she didn’t have any good idea as to what was happening*”* (P14, male, aged 60‐69 years, >10 years in community). While this reliance on family interpreters ensured patients could understand their care, it also created dependence on family members, sometimes leading to delays or added burdens. Participants also noticed changes over time, including greater availability of translators and culturally diverse staff. As one shared, “now they have translators everywhere, even in the parking. Greeters in the hospital who speak their language” (P17, female, aged 30‐39 years, >10 years in community). Another noted, “everywhere there are translators in hospitals” (P18, male, aged 70‐79 years, >10 years in community).

#### Language-Concordant Care

Participants expressed strong appreciation for health care staff who spoke their language, emphasizing that this reduced stress and improved trust. As one noted, “we prefer to go to [specific hospital] just because the nurses and all the other staff member do speak Panjabi. So it’s easier for my grandparents” (P11, female, aged 19‐29 years, >10 years in community). Another explained, “if the person is from South Asian communities and they are feeling shy or hesitating to say something in front of a White person, a South Asian doctor or nurse can help” (P5, male, aged 19‐29 years, 1‐3 years in community). Such accounts highlight how language-concordant care fosters both clarity and cultural comfort.

### Cultural and Religious Needs Not Met

Participants frequently described hospital environments that did not align with their cultural or religious values. These included concerns about gender concordance with providers, lack of culturally appropriate food, and perceived insensitivity to religious practices.

#### Gender-Concordant Care

Gender of providers was an important factor for some participants, particularly for women from Muslim and Sikh backgrounds. One explained, “for me as a Muslim lady, I don’t want anybody to touch me if he’s a man and get naked in front of him while there is another lady available” (P20, female, aged 60‐69 years, >10 years in community). Another recalled, “she would have preferred a female nurse, but they had a male” (P1, male, aged 19‐29 years, >10 years in community). These accounts underscore how gender concordance shaped comfort and dignity in care.

#### Dietary and Religious Respect

Food was another area where participants felt their needs were not adequately met. Some reported being served meals that conflicted with their dietary or religious practices. For instance, 1 participant explained, “she was a very strict vegetarian, but they fed her beef” (P1, male, aged 19‐29 years, >10 years in community). Others described hospital food as unappealing or culturally inappropriate:

It’s a challenge because the hospital food is a challenge for them. They don’t like it, obviously, because it’s not their cultural food.

[P17, female, aged 30‐39 years, >10 years in community]

#### Cultural Insensitivity

In some cases, participants felt their cultural or religious practices were disrespected. One recalled, “she had her religious books and statues by her bed and they moved to a corner by garbage, which is offensive to our religious practices” (P1, male, aged 19‐29 years, >10 years in community). These incidents, though not universal, left lasting negative impressions and diminished participants’ sense of respect for care. Participants recounted instances where staff failed to accommodate dietary and religious needs, sometimes in ways that were profoundly disrespectful.

### Family and Caregiver Roles

Family members were described as essential to the hospital experience, serving as translators, advocates, and sources of emotional support. Yet participants also reported that hospital policies and circumstances sometimes limited family involvement, creating both barriers and additional burdens.

#### Restrictions on Family Presence

Some participants described frustration with restrictions on family members accompanying patients, even when needed for support or translation. One participant recalled, “they were not allowing my family members to come and sit with me” (P2, male, aged 19‐29 years, <1 year in community). Another shared, “sometimes the person needs emotional support or they need someone from the family...but I can’t go with her because they don’t have enough space” (P5, male, aged 19‐29 years, 1‐3 years in community). Such restrictions were perceived as leaving patients vulnerable and isolated.

#### Emotional and Practical Burden on Families

While family involvement was crucial, it often created strain. One participant described, “her son would take her, but he would have to take time off work in order to take her” (P4, female, aged 60‐69 years, >10 years in community). Another reflected on balancing childcare with caregiving: “sometimes I have to be without food for long times...I cannot also go to the cafeteria with kids” (P9, female, aged 30‐39 years, 4‐6 years in community). These stories illustrate how the health system’s reliance on families carries significant costs.

#### Preference for Family Caregiving

While family involvement can be burdensome, many participants preferred it over professional care for trust and cultural reasons. As 1 participant explained, “South Asian people...are more comfortable with their family member, rather than a nurse, doing certain tasks” (P17, female, aged 30‐39 years, >10 years in community). This highlights how family involvement was not only necessary but often desired.

### Discrimination and Bias

Although many participants spoke positively of their care, some described experiences of stereotyping, dismissal, or unequal treatment.

#### Stereotyping and Assumptions

A few participants reported feeling judged on the basis of cultural stereotypes. One explained, “because they’re Indian, they obviously eat unhealthy and because of that they have high blood pressure and heart issues...without doing further research” (P1, male, aged 19‐29 years, >10 years in community). Such assumptions left participants feeling unseen and unfairly blamed.

#### Unequal Quality of Care

Others felt that seniors and immigrants received less attentive or less culturally appropriate care. As one participant described, “I’ve seen that personally with my grandma, that they don’t take seniors that seriously” (P11, female, aged 19‐29 years, >10 years in community). Another reflected, “it’s not very culturally appropriate...my grandparents’ experience is not the same” (P1, male, aged 19‐29 years, >10 years in community). These perceptions shaped trust in the system and influenced decisions about where and when to seek care.

### Positive Experiences and Gratitude

Despite systemic and cultural challenges, participants consistently recognized skilled, compassionate care. One said,

The staff was very good. They were peacefully talking. The doctor was very nice. It was very good.

[P18, male, aged 70‐79 years,>10 years in community]

Another reflected, “I have the best experience and I was very happy” (P3, male, aged 40‐49 years, 1‐3 years in community). Additionally, trust in providers’ skill was often expressed as gratitude, even when other barriers were present. For instance, 1 parent stated, “I’m very grateful to all the doctors and nurses who provided care to my child” (P12, female, aged 40‐49 years, <1 year in community). Such reflections reveal the duality of participants’ experiences: systemic and cultural barriers coexisted with respect for the expertise and dedication of individual providers.

### Perceptions and Expectations of Hospital-at-Home

#### Overview

Participants shared diverse perspectives on virtual hospital services, highlighting benefits, limitations, and conditions under which such care is acceptable. Five major themes emerged—convenience, comfort, and family support; cultural and generational differences; quality of care and clinical limitations; emotional safety, privacy, and trust; and barriers to access and technology. Participants’ experiences and expectations reflected both practical considerations, such as travel and technology, and cultural and interpersonal factors influencing acceptance. Views often diverged across age, gender, and immigration status, with younger or Canadian-born participants more enthusiastic about virtual care and elders or newcomers expressing hesitation.

#### Convenience, Comfort, and Family Support

##### Overview

Participants consistently highlighted the practical and emotional advantages of receiving care at home. Virtual hospital services were valued for reducing stress, increasing comfort, and enabling family involvement, which was seen as supportive both physically and emotionally. Many participants described how being in a familiar environment could enhance recovery, while also reducing exposure to illness compared with hospital settings.

##### Avoiding Travel and Wait Times

Many participants appreciated the time-saving and stress-reducing aspects of virtual care. One noted,

I can be at home and go about my daily things and just wait for a phone call. I don’t have to actually sit at all at an actual office anyway.

[P6, male, aged 40‐49 years, >10 years in community]

Others highlighted avoidance of transportation challenges and hospital stress, stating it would allow them “to get help like at home...I don’t have to stuck in the traffic and get stressed out” (P10, female, aged 60‐69 years, >10 years in community) and “virtual hospitals would be more convenient. He won’t have to worry about transportation waiting. Time to come and go” (P7, male, aged 60‐69 years, 1‐3 years in community).

##### Healing at Home

Being at home with family and in a familiar environment was viewed as beneficial for recovery. Participants described emotional and physical advantages: “you are around your family, that motivates you to be more good. I think that’s the biggest thing that virtual can provide” (P2, male, aged 19‐29 years, <1 year in community), and “if this virtual hospital help was available, then definitely he would have felt more better at home rather than at the hospital...we can read books for him, we can sit beside him” (P17, female, aged 30‐39 years, >10 years in community). Others emphasized comfort and food preferences, noting,

I feel more comfortable in my space...Benefits I can be with my family. That’s the best benefit I can eat the food that I want.

[P16, female, aged 30‐39 years, <1 year in community]

##### Reduced Exposure to Illness

Participants recognized the safety advantage of avoiding hospital-acquired infections: “It can help many people...rushing to the hospitals because of. danger of getting communicable diseases” (P12, female, aged 40‐49 years, <1 year in community), and “with the virtual, then there wouldn’t be so many people waiting for beds. hospitals have a lot of diseases going around” (P15, female, aged 19‐29 years, >10 years in community).

##### Smooth Transitions from Hospital to Home

Several participants highlighted the importance of smooth transitions from hospital to home, emphasizing clear communication, rapid response in emergencies, and reliable monitoring. The availability of equipment and the teach-back style training were viewed as critical.

...how the South Asian will be educated...equipment will be there, they’ll teach you how to use it. It’s important that you use it properly.

[P4, female, aged 60‐69 years, >10 years in community]

If something gets worse, who do I call first? That should be very clear.

[P15, female, aged 19‐29 years, >10 years in community]

##### Home-Based Challenges

Participants noted that home environments can pose challenges during recovery, such as stairs, inaccessible bathrooms, or absence of hospital equipment:

My bedroom is upstairs and the bathroom is downstairs. After surgery, how can I manage the stairs?

[P7, male, aged 60‐69 years, 1‐3 years in community]

We don’t have special hospital beds at home. Sometimes you need that equipment to recover properly.

[P12, female, aged 40‐49 years, <1 year in community]

Routine household responsibilities, such as childcare, also limited the ability to rest: “I know if I go home, I have to cook for the other two children and I have to take care of them” (P20, female, aged 60‐69 years, >10 years in community). Participants worried that home-based care could shift responsibilities onto family members, especially women and elders:

Yes, absolutely, definitely. When I am at the hospital, I’m just quite confident that I’ve been taken care of 100%. But if I am at home, it will put more pressure on me

[P10, female, aged 60‐69 years, >10 years in community]

### Cultural Norms and Generational Differences

#### Overview

Cultural norms, family expectations, and generational comfort with technology strongly influenced participants’ perceptions of virtual hospital care. While younger participants reported ease with online platforms, older adults often struggled with technology and valued in-person monitoring. Cultural beliefs regarding family roles and appropriate care also shaped attitudes toward remote health care.

#### Generational Divide in Comfort with Technology

Participants highlighted that younger generations were more comfortable with technology, while older adults often struggled. One reflected,

For me, growing up in this generation, I’m not shy for technology, but for example, my grandparents, it’s very a new world to them. So for them there is more of that disconnect.

[P1, male, aged 19‐29 years, >10 years in community]

Similarly, a participant noted, “if I imagine my grandmother using this service, she might be overwhelmed with the tablet thing...she would just be like, I just wanna meet people in real” (P16, female, aged 30‐39 years, <1 year in community).

#### Cultural Norms and Expectations of Care

Some participants described South Asian cultural preferences for in-hospital monitoring, emphasizing family support and safety:

I believe for the South Asian people this will not work very well...I would say at the hospital, I feel the care is around me 24 hours...So I think at the hospital we can get the maximum care.

[P20, female, aged 60‐69 years, >10 years in community]

Others stressed the importance of culturally sensitive approaches, including language support: “I think having that community-centered culturally sensitive approach to healthcare is really important because this generic advice doesn’t help the community*”* (P1, male, aged 19‐29 years, >10 years in community).

#### Absence of Support Persons at Home and Immigrant Social Networks

Some participants identified the absence of support persons at home as a barrier to virtual care. As immigrants, many noted that they no longer have the robust support systems they relied on in their native countries. While they can manage their daily lives, participants expressed doubts about their ability to maintain the same level of self-care when unwell, particularly those living alone or with family members who work full time.

It’s very hard because you don’t have much people around you...You don’t have a community...You don’t have neighbors as we use to have uh back in in Asia or South East Asia, there’s a lot of people around you...But here, because we are immigrant, it’s very limited people that we know and it’s not the bond even it’s not that because of the type of living here as we all work or just us to take care of the family...So our relationship is very limited.

[P20, female, aged 60‐69 years, >10 years in community]

### Quality of Care and Clinical Limitations

#### Overview

While participants acknowledged the convenience of virtual care, many expressed concerns about clinical quality. The inability to conduct physical examinations and the perception that remote care could be “lesser” than in-person attention were recurring issues. Participants suggested virtual care might be suitable for routine or minor issues, but not for serious medical concerns.

#### Value of Physical Examination

Many participants expressed concern that virtual care could not substitute for hands-on assessment:

In my view...the Virtual Hospital will not be very effective if the patient is not present in front of the doctor...virtual care will be only communication.

[P3, male, aged 40‐49 years, 1‐3 years in community]

My knee problem...I wasn’t sure how I could show my knees over the phone?

[P13, female, aged 60‐69 years, <1 year in community]

#### Perceptions of “Lesser Care”

Some participants worried that virtual hospital services could be perceived as inferior, noting, “I just feel like if it’s online like people would think like, oh, you’re not taking me seriously” (P11, female, aged 19‐29 years, >10 years in community) and “they wouldn’t fully be supportive...they would think ohh I would receive better care at a hospital rather than me taking my medications on my own” (P15, female, aged 19‐29 years, >10 years in community). Participants suggested virtual care is appropriate for routine or minor concerns, while serious conditions require in-person attention:

If it’s a small thing, then Virtual Hospital is better. But if it’s like a big issue, then she would prefer in-person care.

[P4, female, aged 60‐69 years, >10 years in community]

### Emotional Safety, Privacy, and Trust

#### Overview

Emotional well-being, privacy, and trust emerged as key considerations. Participants emphasized the importance of empathy and interpersonal connection, while also noting concerns about privacy at home and the potential for fraud or security issues.

#### Need for Interpersonal Connection

Participants emphasized empathy and human touch, even via remote communication:

Having that interpersonal touch as much as you can...sometimes having that personal touch makes them feel better. But through a phone, you don’t really get that personal touch, so still making sure whoever is on the line is caring and kind.

[P1, male, aged 19‐29 years, >10 years in community]

#### Concerns About Privacy at Home

Some expressed difficulties discussing sensitive issues at home:

If I want to tell you something, but I don’t want to tell other people at home it could be a concern. So it’s better to go to Doctor [in person].

[P10, female, aged 60‐69 years, >10 years in community]

#### Trust in Fraser Health’s Professionalism

Participants generally assumed privacy and security were managed by a reputable provider: “I would also assume because Fraser Health is a reputed professional organization, they would have already taken all of these requisite steps to make sure their privacy is covered” (P6, male, aged 40‐49 years, >10 years in community), and “I feel like Fraser Health has really good system for privacy” (P11, female, aged 19‐29 years, >10 years in community).

#### Apprehension About Fraud or Scams

Some participants noted the risk of unknown numbers or digital communication: “Only thing is sometimes when you get a call from an unknown number, it seems like it might be a fraud” (P7, male, aged 60‐69 years, 1‐3 years in community).

### Barriers to Access and Technology

Despite interest in virtual care, participants noted practical barriers related to technology and digital literacy. These challenges were particularly salient for older adults and recent immigrants.

#### Digital Literacy Challenges

Older adults may lack experience with technology: “Maybe having like info sessions at temples. helping teach the elders about how it works” (P1, male, aged 19‐29 years, >10 years in community), and “if I imagine my grandmother using this service, she might be overwhelmed with the tablet thing” (P16, female, aged 30‐39 years, <1 year in community).

#### Access to Equipment

Not all households have necessary devices: “Not just like assuming like they have a laptop on them...my mom’s mom has never touched a computer in her life” (P1, male, aged 19‐29 years, >10 years in community).

### Suggestions for Building Awareness of Virtual Hospitals at Home

Participants consistently highlighted the importance of proactive outreach and awareness-building, noting that many newcomers and elders were unaware of existing programs. Outreach strategies were rooted in community-based trust networks, emphasizing cultural familiarity, word of mouth, and visible engagement in South Asian gathering spaces.

#### Leveraging Trusted Spaces and Leaders

Participants recommended outreach through temples, gurdwaras, and mosques, where health authority representatives could host training or information sessions. They stressed that engagement would be more effective if facilitated by respected community leaders.

...meeting with the community leaders and they can like bring it to the community. I know at one of my temples...they have community members teaching them...maybe having someone from Fraser Health going in and doing a training session.

[P6, male, aged 40‐49 years, >10 years in community]

#### Testimonials and Peer Influence

Most participants expressed hesitation about being the first to try the new program, preferring to hear positive testimonials from others before committing. They also mentioned that even a few negative reviews could deter their interest in the program. This reflects the strong sense of mutual trust and reliance on community members, which participants valued just as much as official information provided by the health authority. Hearing positive testimonials from peers was seen as essential to building trust and encouraging adoption.

...having like some kind of testimonial or proven proof that it is already worked before...that would just remove the stress or the doubt. Once it becomes like accepted as a normal thing, that would make it easy.

[P7, male, aged 60‐69 years, 1‐3 years in community]

#### Community Events and Visible Outreach

To engage South Asian communities, participants recommended venues such as libraries, community centers, places of worship, and networking events where community members regularly gather. Additionally, cultural and religious events, such as the annual Vaisakhi Day Parade held in Surrey, were identified as valuable outreach opportunities. Participants suggested setting up information booths equipped with printed materials, visual media, and representatives from the health authority to disseminate information about virtual care in the languages spoken by the community. They noted that organizations providing employment or settlement support (such as PICS) already use these channels effectively, demonstrating their potential for outreach. Furthermore, participants recommended collaborating with local South Asian–based nonprofit organizations.

...we have like festivals of the year, you know Vaisakhi, Culture Fest...maybe having like a Fraser Health pop up so community members can engage with it and educate it that way. Trying to get in the community where you can would be the best.

[P6, male, aged 40‐49 years, >10 years in community]

#### Ethnic Media and Social Platforms

To reach a broader demographic, participants suggested using local South Asian newspapers, radio, or TV networks for individuals to share their lived experiences. This would particularly target the working population who often listen to the radio while driving. Additionally, some participants recommended using social media, a popular platform for news consumption, to further raise awareness.

I think doing more awareness about this virtual...talking with the people, providing more awareness to the people that they should know that such kind of program is existed.

[P7, male, aged 60‐69 years, 1‐3 years in community]

## Discussion

### Principal Findings

This qualitative study explored the experiences of South Asian community members in the Fraser Health region with in-person hospital services, and their perceptions and expectations of Hospital-at-Home models. Participants described significant systemic and cultural barriers in traditional hospital settings, alongside gratitude for providers’ skill and compassion. When discussing Hospital-at-Home, participants articulated both enthusiasm for its convenience and comfort and concerns about quality of care, cultural alignment, and feasibility in their home environments. Taken together, these findings provide important insights for health system planners seeking to advance culturally safe and equitable virtual hospital services.

### Summary of Key Findings

Participants’ hospital experiences reflected 6 key themes: (1) systemic barriers, including long wait times, overcrowding, transportation, and navigation challenges; (2) communication and language barriers; (3) unmet cultural and religious needs; (4) essential but burdensome family and caregiver roles; (5) experiences of discrimination and bias; and (6) positive encounters characterized by gratitude and respect. These issues varied across age, gender, and immigration status, with older and more recently arrived participants reporting more barriers.

Expectations of Hospital-at-Home revealed 5 major themes: (1) convenience, comfort, and family support; (2) cultural and generational differences; (3) quality of care and clinical limitations; (4) emotional safety, privacy, and trust; and (5) barriers to access and technology. Across both sets of findings, participants consistently highlighted the central role of family, the importance of cultural sensitivity, and the need for accessible information delivered through trusted community channels.

Participants’ mixed experiences with in-person hospitals provide essential context for understanding perceptions of Hospital-at-Home. Structural challenges, such as prolonged emergency department waits and overcrowded wards, reduced trust in the timeliness and equity of hospital care. For newcomers, health system literacy gaps compounded these difficulties, as participants struggled to understand coverage, referral pathways, and navigation. These frustrations may increase openness to Hospital-at-Home as an alternative, particularly if it can reduce wait times and enhance comfort. However, systemic inequities that disadvantage newcomers and elders risk being reproduced in virtual formats unless addressed proactively.

### Comparison to Existing Literature

Previous research has extensively documented barriers to virtual health care access for South Asians. Studies by Zibrik et al [18] and Makowsky et al [19] highlight disparities in virtual care access, while Dahal et al [29] and Hyman et al [16] emphasize the impact of socioeconomic factors on virtual health engagement. Notably, few studies have examined Hospital-at-Home programs within other racial or ethnic minority communities, limiting opportunities for direct comparison. This scarcity of literature underscores the novelty of exploring Hospital-at-Home acceptability specifically among South Asian populations in Canada and highlights the unique structural, cultural, and familial considerations identified in this study.

Findings from this study align with existing literature in confirming that language barriers, trust in health care providers, and digital literacy shape perceptions toward adopting virtual care. Moreover, consistent with Bhalla et al [30], community-driven strategies, such as the role of family members in health decision-making and the influence of social networks such as WhatsApp, play a crucial role in health care engagement for the South Asian community. These findings were echoed by participants of this study who noted their attitudes toward Hospital-at-Home care could be highly influenced by peer testimonials.

A key distinction of this study is its focus on Hospital-at-Home models rather than primary care or outpatient virtual services, which have been the emphasis of much prior research. While previous research has explored South Asians’ engagement with digital tools for chronic disease management and primary care, this study surfaces concerns that are specific to the delivery of hospital-level care in the home. In particular, it contributes new insights on the role of the home environment, including the physical suitability of the space, the availability and involvement of family caregivers, the complexities of intergenerational living arrangements, and the burden of household responsibilities during recovery from acute episodes.

These burdens often fall disproportionately on women, particularly mothers, reflecting traditional caregiving roles common in South Asian cultures that persist postimmigration. Female caregivers frequently manage both direct patient care and household duties, reinforcing the gendered division of labor described in previous literature [16]. Recognizing this, Hospital-at-Home programs should incorporate gender-sensitive supports, such as education, respite resources, and guidance for safe patient care, to mitigate additional burdens on women.

Another critical finding relates to the health care system education to improve virtual care access. Dahal et al [29] emphasize the role of community organizations in supporting newcomers through education on health care navigation. Participants in this study similarly reported limited awareness of services such as urgent and primary care centers and the 8-1-1 line, resulting in unnecessary reliance on emergency departments. This highlights that health system literacy is a foundational barrier: without addressing these basic knowledge gaps, new service models such as Hospital-at-Home risk reproducing existing inequities rather than improving access.

Chowdhury et al [31] identified concerns about virtual care creating distance between patients and providers, a theme also present in this study. Participants feared that reduced in-person interactions could diminish care quality and trust. This included concerns about developing weaker relationships with providers due to the impersonal nature of virtual interactions, which participants felt could compromise the depth of care and empathy received. This concern reinforces the importance of maintaining strong communication strategies within Hospital-at-Home models to ensure services remain patient-centered and do not further alienate those who experience barriers to care.

While age, gender, and immigration status emerged as individual factors shaping experiences with Hospital-at-Home, our findings underscore the importance of viewing these dimensions through an intersectional lens. For example, older newcomers with limited English proficiency described significant challenges with both language and digital literacy, creating compounded barriers to adoption that were distinct from those faced by younger, Canadian-born participants who reported greater comfort with system navigation and technology use. Similarly, caregiving responsibilities were disproportionately reported by women in multigenerational households, highlighting how gendered expectations intersect with cultural norms and family structures to intensify the caregiving burden. Recent immigrants, regardless of age, also described limited awareness of alternatives to the emergency department, illustrating how immigration status interacts with health system literacy to shape health care–seeking behaviors. These examples illustrate that barriers to Hospital-at-Home adoption are not experienced uniformly across South Asian communities but rather reflect the cumulative and intersecting effects of multiple social identities. Recognizing these layered experiences is critical for designing flexible, equity-oriented models that can adapt to the diverse needs within South Asian populations.

### Implications for Hospital-at-Home Implementation

Despite substantial evidence demonstrating the effectiveness of Hospital-at-Home programs in improving patient outcomes and reducing health care costs, significant knowledge gaps remain regarding how to scale these services across diverse populations [32]. This study addresses this gap by exploring the adoption barriers and enablers of Hospital-at-Home services among South Asian communities in the Fraser Health region, offering insights for culturally tailored implementation strategies.

Increasing awareness of Hospital-at-Home services in ways that resonate with South Asian communities is essential to enhance willingness to participate. Findings from this study indicate that South Asian communities prefer learning about Hospital-at-Home through trusted channels such as community organizations, cultural events, and peer testimonials. Additionally, benefits such as increased privacy at home, proximity to family members, and greater control over aligning care with cultural and religious practices were identified as factors that enhance the appeal of Hospital-at-Home services for South Asian patients.

From a systems perspective, raising awareness about how Hospital-at-Home fits within the broader health care system is equally important. Educating patients and caregivers about the structure of health care services and how Hospital-at-Home complements traditional hospital care can build trust, increase understanding of its role in acute care delivery, and foster confidence in available escalation pathways. This system-level approach can help patients see Hospital-at-Home as an integral part of a coordinated healthcare continuum rather than as a stand-alone service.

Addressing individual barriers is also critical for sustainable and equitable adoption. Limited digital literacy skills among South Asian populations can be mitigated through multilingual education and training strategies that accommodate diverse learning styles. Designing user-friendly digital interfaces for Hospital-at-Home services is also critical to ensure accessibility. Furthermore, the study highlights the importance of explicitly considering family members and caregivers in Hospital-at-Home implementation. This includes developing caregiver-focused training materials, addressing family dynamics in care planning, and regularly evaluating caregiver experiences.

This study further emphasizes that preferences and attitudes toward Hospital-at-Home services are not uniform across South Asian communities, nor do they universally apply to all patient needs. For instance, while some participants appreciated the comfort and cultural familiarity of home-based care, others voiced significant concerns around privacy, data security, and the adequacy of clinical care delivered virtually. These diverging views underscore the need for flexible, patient-centered models that allow for individual choice.

Housing environments emerged as an important contextual factor influencing the feasibility of Hospital-at-Home care. Participants described challenges such as stairs, limited space for medical equipment, and crowded or multigenerational households, all of which could constrain the safety and acceptability of receiving acute care at home. These findings point to the need to consider how physical living environments intersect with the delivery of virtual hospital models. While a detailed policy analysis is beyond the scope of this study, it is important to acknowledge that housing affordability and adequacy are pressing issues in Canada, particularly in urban centers such as Surrey. Future research should explore how these broader structural determinants, including housing conditions, shape equitable access to home-based care models and whether supportive housing or community infrastructure can mitigate such barriers.

To ensure equitable and effective implementation, the design of Hospital-at-Home programs must incorporate sufficient resources to continuously engage diverse patient, provider, and community voices. This ongoing feedback process is critical for adapting services to reflect the cultural nuances, varied needs, and evolving preferences of South Asian populations, enabling the development of culturally informed strategies that foster sustained adoption and trust in Hospital-at-Home services. This study’s qualitative findings informed a subsequent Experience-Based Co-Design initiative, which actively engaged South Asian community members in the cocreation of culturally responsive solutions to the challenges identified. Specific outcomes of this process included the development of culturally adapted training materials, multilingual communication tools, and revised care workflows to strengthen trust and clarity in care delivery. By linking this foundational research with participatory design methods, the broader project not only addressed knowledge gaps but also demonstrated a model for translational research, showing how community-engaged approaches can directly inform the design of culturally safe virtual care innovations.

### Policy and System-Level Considerations

The findings from this study highlight the need for equity-focused policies and system-level interventions to enhance virtual care accessibility for South Asian communities in Fraser Health and beyond. A central implication is that health system literacy must be addressed as a prerequisite to virtual adoption; simply introducing Hospital-at-Home services without parallel education on how and when to use them may not improve access. South Asian patients may face barriers due to limited English proficiency and a lack of access to culturally competent care [33]. Policies should mandate the integration of multilingual virtual health services, including real-time interpretation and culturally responsive health communication strategies. This aligns with Fraser Health’s ongoing efforts to improve accessibility through initiatives such as the SAHI.

Another critical policy recommendation is the standardization of digital literacy support within virtual care services. The findings of this study identified the problematic impact of technological barriers, particularly among older adults and individuals unfamiliar with digital tools. Policies should ensure that virtual care platforms are user-friendly and that comprehensive digital literacy training programs are offered as part of virtual hospital enrollment. This could include step-by-step tutorials, one-on-one technical assistance, and hybrid learning options that combine online and in-person training [34]. In addition, although only a minority of participants mentioned it, data privacy and security remain essential considerations in the design of virtual hospital services. Policies should ensure that multilingual and culturally tailored digital platforms also meet high standards of technological ethics, including secure data management and patient confidentiality, to build and maintain trust among diverse communities.

System-level reforms should focus on streamlining care pathways for virtual hospital patients. Participants expressed concerns about delays in care escalation and emergency response times. To address this, policies should define clear transition protocols between virtual and in-person hospital care, ensuring that patients requiring higher levels of medical attention can be seamlessly transferred. Emergency response times can be optimized through virtual triage systems to ensure virtual hospital patients receive timely interventions when needed [35] .

An additional consideration is the inclusion of family caregivers in virtual health planning. Individuals in South Asian households rely on family members and loved ones for health care navigation and support. Virtual care policies should recognize and integrate the role of caregivers, facilitating access to training, resources, and decision-making tools to support patient care effectively.

Finally, data-driven policy improvements are essential for ensuring that virtual care models address the specific needs of South Asian communities. These findings of this study highlight the importance of ongoing patient engagement and feedback mechanisms. There is a clear need to implement structured monitoring and evaluation frameworks that assess adoption, patient experiences, health outcomes, and engagement levels in virtual care programs. These insights should inform policy refinements to ensure equitable and effective virtual health services.

### Strengths and Limitations

This study provides novel insights into Hospital-at-Home acceptability among South Asian communities in Canada, a population underrepresented in Hospital-at-Home research. Semistructured interviews in multiple languages enabled diverse perspectives across gender, age, and immigration status. Importantly, the study included voices of marginalized groups—such as elders, recent immigrants, and family caregivers—who are often excluded from virtual health research.

While this study provides valuable insights into the experiences of South Asian communities with virtual health care, several limitations should be noted. Recruitment challenges limited geographic diversity, with most participants residing in Surrey. As access to health services and digital infrastructure varies across municipalities, future studies should aim for broader regional representation to capture a wider range of experiences.

Another limitation is the reliance on referrals from PICS, which may have introduced selection bias. Participants recruited through PICS are likely to have stronger community connections and greater awareness of health resources, leading to a sample more engaged with the health care system than the broader South Asian population. This may result in an underrepresentation of those facing greater systemic and socioeconomic barriers. Nonetheless, the inclusion of participants with limited English proficiency, lower digital literacy, and newcomer status partially mitigates this concern by ensuring that marginalized perspectives were represented.

The reliance on online interviews is another limitation. While virtual interviews increased accessibility for some, they may have excluded participants uncomfortable with digital technologies or lacking reliable internet access, potentially missing the perspectives of those most affected by digital exclusion. Future research should incorporate alternative data collection methods, such as in-person or telephone interviews, to ensure more inclusive participation.

Despite these limitations, this study offers valuable insights into the structural and cultural barriers influencing virtual health adoption among South Asian communities. Future research should use diverse recruitment strategies and mixed methods approaches to enhance sample representativeness and ensure that equity-driven virtual care models are more inclusive of underserved populations.

### Conclusion

This study highlights the complex and context-specific factors that influence South Asian communities’ engagement with Hospital-at-Home and virtual care services. Barriers such as limited digital literacy, language challenges, caregiver burden, and lack of awareness about health care alternatives must be addressed through culturally and linguistically responsive strategies. At the same time, key facilitators such as trust in health care providers, the comfort of home-based care, and alignment with cultural and familial values offer important starting points for building more inclusive models of care. Ensuring the equitable implementation of Hospital-at-Home services will require sustained investment in community-specific education, caregiver inclusion, and ongoing feedback mechanisms that reflect the diversity within South Asian populations.

## Supplementary material

10.2196/79675 Multimedia Appendix 1 Full interview guide.

10.2196/79675 Checklist 1 COREQ checklist.

## Abbreviations

PICS: Progressive Intercultural Community Services
SAHI: South Asian Health Institute
