# Supplementary material for: Hospital-at-Home for South Asian Communities in British Columbia, Canada: Qualitative Interview Study
Source: JMIR Hum Factors. 2026 Jan 5;13:e79675. doi: 10.2196/79675 (PMC12768396; doi:10.2196/79675)
Supplement: Multimedia Appendix 1 [file humanfactors-v13-e79675-s001.docx]

# Multimedia Appendix 1: South Asians For Equitable (SAFE) Virtual Care: Interview Topic Guide

If you would like me to repeat a question, please let me know. We encourage you to take all the time you need before answering a question. We can also return to a question later if you would like more time to think about it. When sharing your responses to questions in the interview, please consider from the perspective of the South Asian community.

May I ask if you identify yourself primarily as a patient (who receives care), or a caregiver (who regularly looks after another individual receiving care)?

Patient/Caregiver Experience with In-Person Hospital Care:

1. As a member of the South Asian community, please tell us about your experience getting care in the hospital, either as a patient or caregiver?
2. Have you seen or heard about any challenges or barriers to getting hospital care by members of the South Asian community?
   - If yes, can you please describe
3. What do you think would make it easy for South Asian community members to use hospital services?
   - *Alternative wording:* Are there things that you think might help other South Asian community members access hospital services?
4. How has distance, travel, or transportation affected your or others’ ability to get to the hospital?
5. Does being in the hospital affect your ability to stay connected with your caregivers or family members?
   - If yes, please describe.
6. If we could improve only one or two parts of your hospital experience, what would they be?
   - *Alternative wording:* if you were in charge, what are one or two things about your hospital experience you would choose to improve first?

Patient Experience, Perceptions, and Attitudes towards Virtual Hospitals:

(*[Play Virtual Hospital Introduction Video](https://app.vyond.com/videos/89f4cf10-f8bb-46fb-8803-8f83e4299c6e)*) ~ 3 minutes

We are creating a Virtual Hospital service. A Virtual Hospital is another way for people to get medical care that they would normally get in the hospital. Instead, a person can get that medical care in the comfort of their own home. People using the Virtual Hospital are considered ‘admitted’ people in the hospital. We provide care mostly at a distance, using virtual technology. The care team will visit people in their home when needed to assess or treat their health condition. A person has a video visit with their health care team at least two times a day to assess their health and healing.

People would be monitored remotely using technology such as tablets linked with information from medical devices like blood pressure machines. People would be able to connect with their health care team 24 hours a day using these technologies – for example by phone, video or even chats, and their care teams to communicate and share information about their health. The care team would be similar to that in the hospital, doctors, nurses and allied health. What questions do you have about the Virtual Hospital before we continue with the interview?

Fraser Health serves the largest South Asian population in BC. As we are creating this Virtual Hospital service, we would like to learn more about how we can better serve the unique needs of this community.

1. Have you or your family used any virtual health services in the past? What was your experience like using these services?
   - For example, having a virtual visit with your health care provider over the phone or a video call, or having your health monitored from home by your health care team
2. Do you feel that there are any differences in quality between in-person and virtual health services? If so, why?
   - *Optional:* What are some ways you think that we could change that (or bridge that gap in quality)?
3. What are some ways you think the Virtual Hospital could help with the challenges faced by the South Asian community in getting healthcare?
4. What would matter the most to you in a Virtual Hospital care setting?
5. How open would you be to using a Virtual Hospital service at home instead of staying in the hospital? Please share why or why not?
   - Alternative wording: If your doctor at the hospital suggests you use our Virtual Hospital service - what would be your reaction?
6. What do you think are some of the possible benefits of using the Virtual Hospital Service for you or your family?
7. What concerns would you have about using the Virtual Hospital Service for you or your family?
8. What concerns might you have about your privacy if using the Virtual Hospital service?
9. We want share more information about the Virtual Hospital service with the South Asian community. What are some ways we could help people learn more about our Virtual Hospital service?
10. If you wanted to use the Virtual Hospital service, how can we make it as easy as possible for you to use it?

Closing:

1. What final thoughts would you like to share about the idea of a virtual hospital?
